# Prognostic value of serial neutrophil-to-lymphocyte ratio measurements in hospitalized community-acquired pneumonia

**DOI:** 10.1371/journal.pone.0250067

**Published:** 2021-04-15

**Authors:** Heock Lee, Insu Kim, Bo Hyoung Kang, Soo-Jung Um

**Affiliations:** Department of Internal Medicine, Pulmonology Division, Dong-A University Hospital, College of Medicine, Dong-A University, Busan, Republic of Korea; Heidelberg University Hospital, GERMANY

## Abstract

**Introduction:**

Several serum inflammatory markers are associated with poor clinical outcomes in community-acquired pneumonia (CAP). However, the prognosis and early treatment response in hospitalized CAP patients based on serial neutrophil-to-lymphocyte ratio (NLR) measurement has never been investigated.

**Methods:**

We performed a retrospective observational study for 175 consecutive patients hospitalized with CAP between February 2016 and February 2018. NLR, C-reactive protein (CRP) and procalcitonin levels were measured on admission day (D1) and on hospital day 4 (D4). The Pneumonia Severity Index (PSI) was also assessed on admission. The primary endpoint was all-cause death within 30 days after admission. The secondary endpoint was early treatment response such as intensive care unit (ICU) admission during hospitalization and clinical unstability on day 4.

**Results:**

The 30-day mortality rate was 9.7%. In multivariate analysis, NLR D4 (OR: 1.11; 95% CI: 1.04–1.18; *P* = 0.003) and its incremental change (NLR D4/D1 >1) (OR: 7.10; 95% CI: 2.19–23.06; *P* = 0.001) were significant predictors of 30-day mortality. NLR D4 and its incremental change were significant predictors of ICU admission and clinical unstability on day 4 in multivariate analyses. Adding of incremental NLR change significantly improved the prognostic ability of the PSI. The additive value of incremental NLR change for the prognostic ability of the PSI was larger than that of incremental CRP change.

**Conclusion:**

Serial NLR measurement represents useful laboratory tool to predict the prognosis and early treatment response of hospitalized CAP patients.

## Introduction

Community-acquired pneumonia (CAP) is major cause of morbidity and mortality in developed countries [1]. Early risk stratification and appropriate treatments are essential to improve CAP prognosis. Either the pneumonia severity index (PSI) [2] or CURB-65 [3] has been widely used in initial assessment to help physicians make more rational decisions regarding CAP severity and prognosis [4, 5]. However, these traditional scoring systems for the pneumonia severity do not give enough information to assess early treatment response and are complicated to measure in hospitalized CAP patients [6].

Many investigators have tried to determine the prognostic value of serum inflammatory biomarkers such as white blood cell (WBC) and its subtypes, C-reactive protein (CRP), and procalcitonin (PCT), interleukin-6, interleukin-8, interferon-alpha, and tumor necrosis factor in CAP patients. Although WBC count, CRP, and PCT measured on admission were useful in early risk stratification, their predictive role remains controversial [7–10]. Furthermore, some of these markers have limitations attributable to its relatively high cost and low accessibility, so there is still a need for simple, specific, readily available and not expensive biomarkers in CAP patients.

Recently, several studies showed that serial CRP and PCT measurements had more predictive value than one-time testing of these markers [11–13]. The neutrophil-to-lymphocyte ratio (NLR), which is the absolute neutrophil count divided by the absolute lymphocyte count, is a readily available laboratory marker used to evaluate infectious diseases [14]. One-time NLR measurement has been demonstrated as a useful marker to assess the severity and predict the prognosis of CAP patients [15–17].

Herein, we sought to evaluate the value of serial NLR measurements in predicting the prognosis and assessing early treatment response in hospitalized CAP patients, and the benefit of adding serial NLR measurement to the PSI.

## Methods

### Study design

This retrospective cohort study included consecutive CAP patients hospitalized at Dong-A University Hospital from Feb 2016 to Feb 2018. According to the Infectious Diseases Society of America (IDSA)/American Thoracic Society (ATS) guidelines [18], CAP was defined by the presence of new infiltrates on chest radiograph and at least one of the clinical symptoms (cough, sputum, shortness of breath, fever, pleuritic chest pain, and hemoptysis) without recent hospitalization. Outpatient follow-up data were reviewed to ensure that the discharge diagnosis was considered to be CAP. Exclusion criteria were change to other diagnoses like pulmonary edema, pulmonary embolism, pulmonary tuberculosis or lung cancer after hospital discharge, antibiotics treatment for more than 24 h at the time of enrollment, absence of WBC differential counts, and conditions known to affect total WBC and its subtype counts such as haematologic disorders, current steroid use, history of steroid therapy within 3 months before current admission, history of radiotherapy or chemotherapy within 4 weeks before enrollment. The severity of pneumonia was assessed with PSI [2] on the day of admission. Our study was approved by Dong-A University institutional review board (DAUHIRB-20-080) and was conducted in accordance with the Declaration of Helsinki. Dong-A University institutional review board waived the requirement for informed consent.

### Inflammatory biomarkers

Total WBC and its subtypes, CRP, and PCT were measured on admission before antibiotics treatment (D1) and on hospital day 4 (D4). The NLR was defined as the absolute neutrophil count divided by the absolute lymphocyte count. Total WBC, neutrophil, and lymphocyte counts were determined using a Sysmex XE-2100 Hematology Analyzer (Sysmex Corp., Kobe, Japan). The serum CRP level was determined with an automated latex-enhanced turbidimetric immunoassay (TBA-200FR; Toshiba, Tokyo, Japan) within 1 h of sample collection. The PCT level was determined using a Vidas BRAHMS PCT enzyme-linked fluorescent immunoassay (BioMerieux, France).

### Study endpoints

The primary endpoint of the current study was all-cause death within 30 days after index admission. The secondary endpoint was early treatment response determined with intensive care unit (ICU) admission during index hospitalization and failure to attain clinical stability on hospital day 4. Clinical stability was defined using the IDSA/ATS guideline criteria [18]. Patients were regarded as clinically stable when all the following criteria were met: body temperature below 37.8°c, heart rate below 100 beats/min, respiratory rate below 25 breaths/min, systolic blood pressure above 100 mmHg, arterial oxygen saturation above 90% or PO_2_ above 60 mmHg on room air, normal mental status, and ability to maintain oral intake.

### Statistical analysis

Continuous variables are shown as median and interquartile range or mean and standard deviation, as appropriate. Differences between continuous variables were determined using the Mann-Whitney test. Categorical variables are presented as frequencies. Differences in categorical variables were analyzed with the Pearson’s chi-square test or the Fisher’s exact test. We used univariate and multivariate logistic regression analysis to assess the role of systemic inflammatory biomarkers in clinical outcomes adjusting the models for age, sex and PSI class. Survival curves were generated using the Kaplan-Meier method, and the difference between curves was assessed by the Log-Rank test. The discriminative abilities of each variable in predicting 30-day mortality or ICU admission were determined with the receiver operating characteristic (ROC) curve analysis and area under the curve (AUC) values. The final ability of the multivariate model was analyzed using the Harrell’s C-statistics. Comparisons between the AUCs were performed with the Hanley and McNeil test. A P value less than 0.05 was considered statistically significant. All statistical analyses were conducted with SPSS 25.0 (SPSS Inc., Chicago, IL, USA) and MedCalc 19.2.0 (MedCalc Software Ltd, Ostend, Belgium).

## Results

### Study population

**Fig 1:**
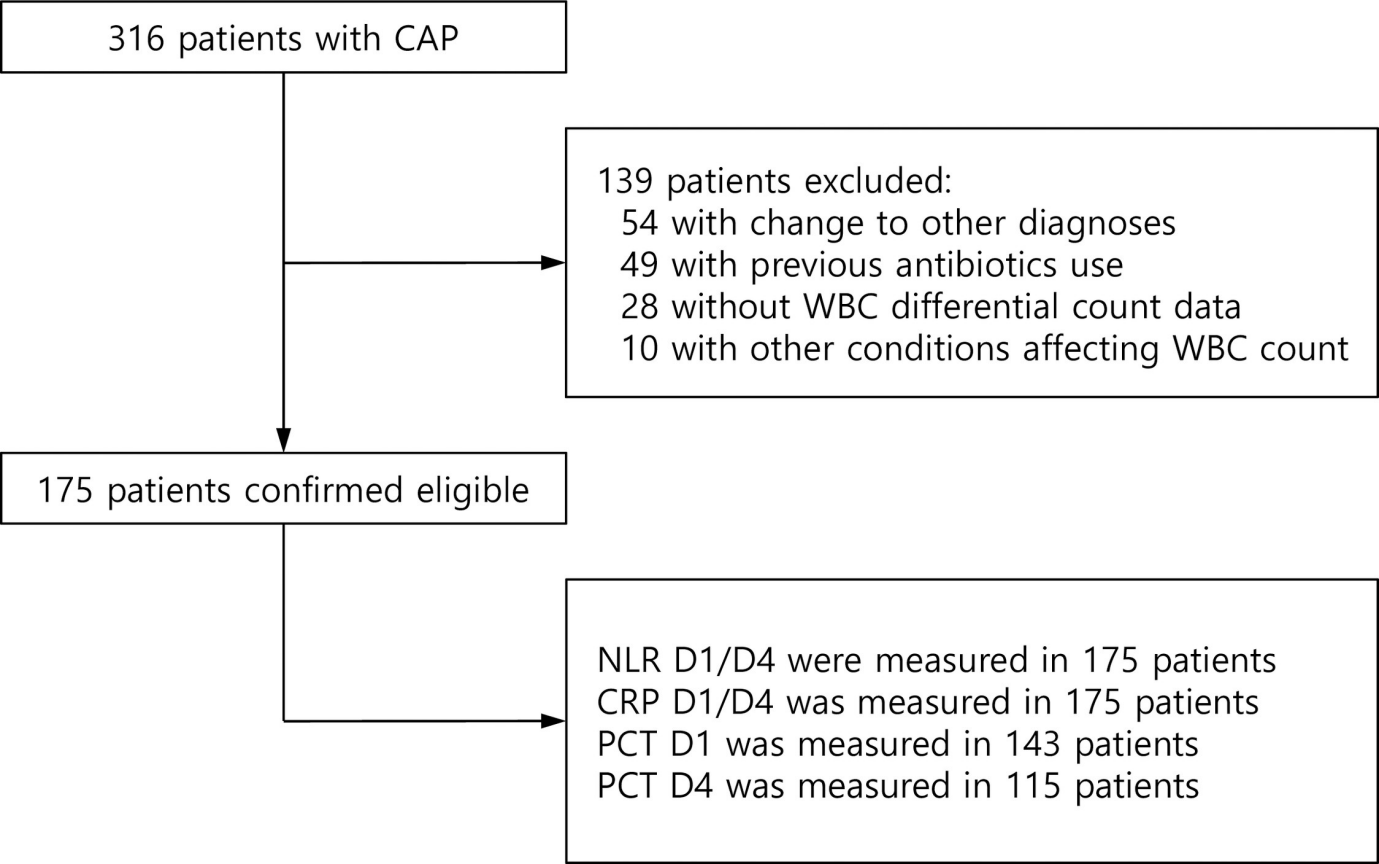
Study flow diagram.

Of the total 316 patients diagnosed with CAP, 175 were eligible for our analyses (Fig 1). The baseline clinical characteristics are presented in Table 1. The mean age was 67.7 ± 12.3 years and 70.9% of patients were men. Twenty-three (13.1%) patients were admitted to ICU and 17 patients died within 30 days after admission (30-day mortality rate: 9.7%). The causative pathogens were identified in 62 (35.4%) patients. The most common microorganisms were: *Streptococcus pneumonia* (11.4%), *Klebsiella pneumonia* (5.1%), *Pseudomonas aeruginosa* (2.9%), *Acinetobacter baumanii* (2.9%), influenza virus (2.3%), *Mycoplasma pneumoniae* (1.7%), Methicillin-resistant *Staphylococcus aureus* (1.1%) and others (4.6%).

**Table 1:** Baseline characteristics of all subjects.

All patients	175
Age, year	67.7 ± 12.4
Men	124 (70.9)
Comorbidities	
Cardiovascular disease	33 (18.9)
Diabetes mellitus	33 (18.9)
Respiratory diseases^a^	32 (18.3)
Malignant disease	21 (12.0)
Liver disease	7 (4.0)
Chronic kidney disease	8 (4.6)
PSI, class	
I	0 (0)
II	55 (31.4)
III	47 (26.9)
IV	50 (28.6)
V	23 (13.1)
SOFA on admission, score	
0–1	63 (36.0)
2–3	66 (37.7)
4–5	30 (17.1)
≥6	16 (9.1)
ICU admission	23 (13.1)
Clinical unstability on hospital day 4	43 (24.6)
30-day mortality	17 (9.7)

ICU: intensive care unit; PSI: pneumonia severity index; SOFA: sequential organ failure assessment.

Values are given as means ± standard deviation or numbers (%).

^a^Respiratory diseases indicate chronic obstructive pulmonary disease, tuberculosis destroyed lung, bronchiectasis, bronchial asthma.

### Inflammatory biomarkers and clinical outcomes

Table 2 presents the values of NLR, CRP and PCT, and their serial changes in survivors and non-survivors. NLR D4 and NLR D4/D1 ratio were markedly higher in non-survivors than in survivors. Furthermore, incremental NLR change (NLR D4/D1 > 1) was observed more frequently in non-survivors than in survivors. Among the values and serial changes in CRP and PCT, only the CRP D4 level was significantly higher in non-survivors than in survivors.

**Table 2:** Systemic inflammatory biomarkers in survivors and non-survivors.

	All patients (n = 175)	Survivors (n = 158)	Non-survivors (n = 17)	*P* value
NLR				
D1	10.1 (5.6–18.8)	9.9 (5.5–19.4)	11.5 (5.4–15.3)	0.964
D4	5.9 (3.3–10.5)	5.4 (3.2–9.8)	11.1 (7.8–25.5)	<0.001
D4/D1	0.62 (0.30–1.02)	0.55 (0.30–0.91)	1.29 (0.61–2.72)	0.001
D4/D1 > 1	44 (25.1)	33 (20.9)	11 (64.7)	<0.001
CRP, mg/dL				
D1	11.9 (6.3–22.7)	11.4 (6.1–22.6)	19.8 (12.0–23.8)	0.054
D4	7.2 (3.0–13.1)	6.7 (2.9–11.9)	15.9 (5.1–21.3)	0.005
D4/D1	0.61 (0.35–1.04)	0.61 (0.34–0.97)	0.77 (0.47–1.16)	0.244
D4/D1 > 1	46 (26.3)	39 (24.7)	7 (41.2)	0.121
PCT, ng/mL				
D1	0.95 (0.32–3.59)	0.99 (0.36–3.51)	0.84 (0.23–21.6)	0.854
D4	0.44 (0.15–1.59)	0.41 (0.14–1.44)	0.71 (0.35–8.01)	0.104
D4/D1	0.43 (0.20–0.98)	0.37 (0.19–0.92)	0.74 (0.40–1.09)	0.084
D4/D1 > 1	22 (20.4)	18 (19.4)	4 (26.7)	0.363

CRP: C-reactive protein; NLR: neutrophil-lymphocyte ratio; PCT: procalcitonin

D1, D4, and D4/D1 values are given as medians (interquartile range).

D4/D1 > 1 value is given as numbers (%).

Table 3 shows NLR values in several subgroups. Among the non-survivors, NLR D4 was significantly higher in patients who died within 7 days of hospitalization than in patients who died since 7 days of hospitalization. Among the survivors, NLR D4 and NLR D4/D1 ratio were significantly higher in patients stabilized after 14 days of hospitalization than in patients stabilized earlier. In addition, incremental NLR change was observed more frequently in patients stabilized later than in patients stabilized earlier.

**Table 3:** NLR values in several subgroups.

Subgroup NLR	Bacterial pathogen (n = 55)	Gram (+) bacteria (n = 32)	Gram (-) bacteria (n = 23)	*P* value
D1	11.5 (6.8–21.5)	11.2 (7.1–26.9)	12.0 (6.0–20.4)	0.603
D4	6.9 (4.1–13.0)	6.7 (4.6–13.0)	7.2 (3.0–13.8)	0.932
D4/D1	0.59 (0.30–1.02)	0.62 (0.29–0.99)	0.44 (0.31–1.38)	0.851
D4/D1 > 1	14 (25.5)	7 (21.9)	7 (30.4)	0.539
Subgroup NLR	All non-survivors (n = 17)	Early non-survivors (≤ 7 days) (n = 3)	Late non-survivors (8~30 days) (n = 14)	*P* value
D1	11.5 (5.8–15.0)	15.6 (9.5–18.2)	10.9 (5.8–14.3)	0.591
D4	11.1 (8.1–23.0)	28.8 (24.7–36.9)	9.9 (7.5–22.8)	0.032
D4/D1	1.29 (0.70–2.55)	2.88 (2.14–4.54)	1.08 (0.52–2.49)	0.091
D4/D1 > 1	11 (64.7)	3 (100)	8 (57.1)	0.243
Subgroup NLR	All survivors (n = 158)	Early stabilized patients (≤ 14 days) (n = 107)	Late stabilized patients (> 14 days) (n = 51)	*P* value
D1	9.9 (5.5–19.4)	10.5 (6.3–20.1)	9.0 (4.9–19.1)	0.275
D4	5.4 (3.2–9.8)	4.4 (2.5–9.0)	7.4 (4.3–11.3)	0.001
D4/D1	0.55 (0.30–0.91)	0.50 (0.27–0.73)	0.83(0.49–1.38)	<0.000
D4/D1 > 1	33 (20.9)	13 (12.1)	20 (39.2)	<0.000

NLR: neutrophil-lymphocyte ratio; PCT: procalcitonin.

D1, D4, and D4/D1 values are given as medians (interquartile range). D4/D1 > 1 value is given as numbers (%). NLR: neutrophil-lymphocyte ratio; PCT: procalcitonin.

Compared with patients treated at a general ward, patients admitted to ICU showed significantly higher values of NLR D4 (10.1 [7.4–20.7] vs. 5.0 [3.0–9.7]; *P* < 0.001), CRP D4 (13.4 [5.7–20.6] vs. 6.3 [2.8–11.8]; *P* = 0.001), and higher percentage of incremental NLR change (65.2% vs. 19.1%; *P* < 0.001). In addition, patients who failed to attain clinical stability on day 4 showed significantly higher levels of NLR D4 (10.1 [6.6–20.7] vs. 4.5 [3.0–9.3]; *P* < 0.001), CRP D1 (16.8 [8.3–23.7] vs. 10.4 [5.3–22.1]; *P* = 0.028), CRP D4 (12.4 [4.8–19.8] vs. 5.8 [2.8–11.1]; *P* < 0.001), PCT D1 (2.08 [0.68–16.2] vs. 0.58 [0.30–2.98]; *P* = 0.008), and PCT D4 (0.74 [0.35–4.89] vs. 0.33 [0.12–1.36]; *P* = 0.002) and higher percentages of incremental NLR change (53.5% vs. 15.9%; *P* < 0.001) and incremental CRP change (37.2%, 22.7%; *P* = 0.049) than patients who were clinically stable on day 4. Patients with incremental NLR change were hospitalized longer than patients without incremental NLR change (21.7 ± 18.1 days vs. 13.7 ± 11.3 days; *P* < 0.001).

### Predictors of 30-day mortality

**Fig 2:**
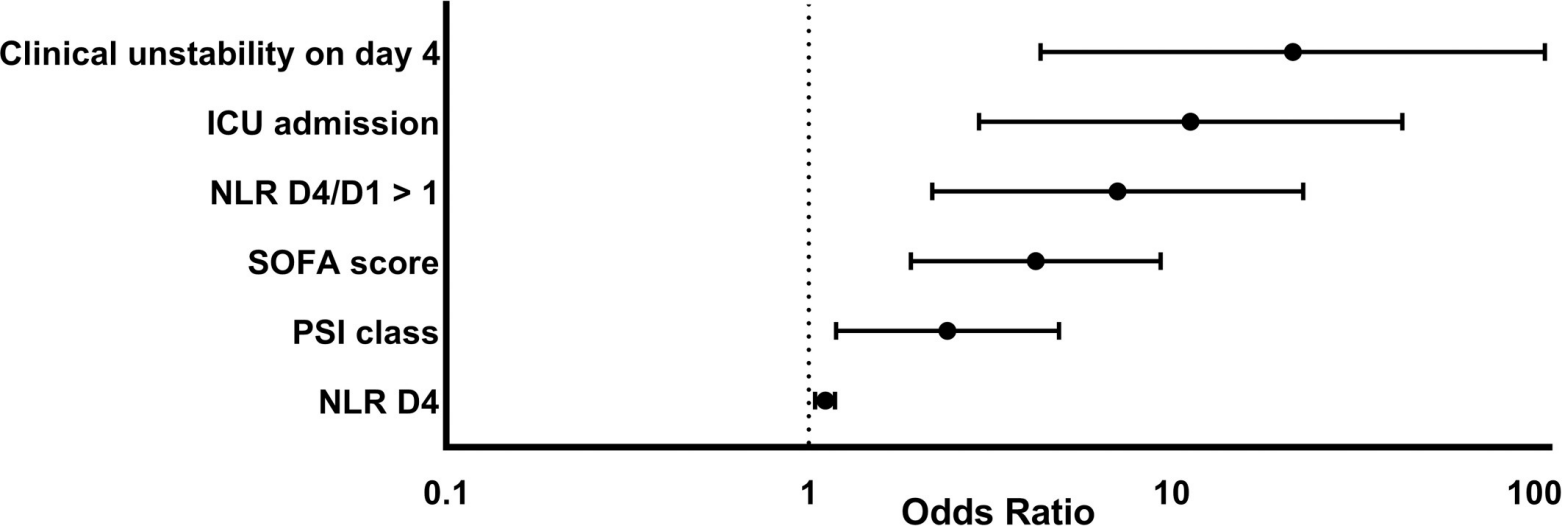
Odds ratios of several parameters to predict 30-day mortality.

**Fig 3:**
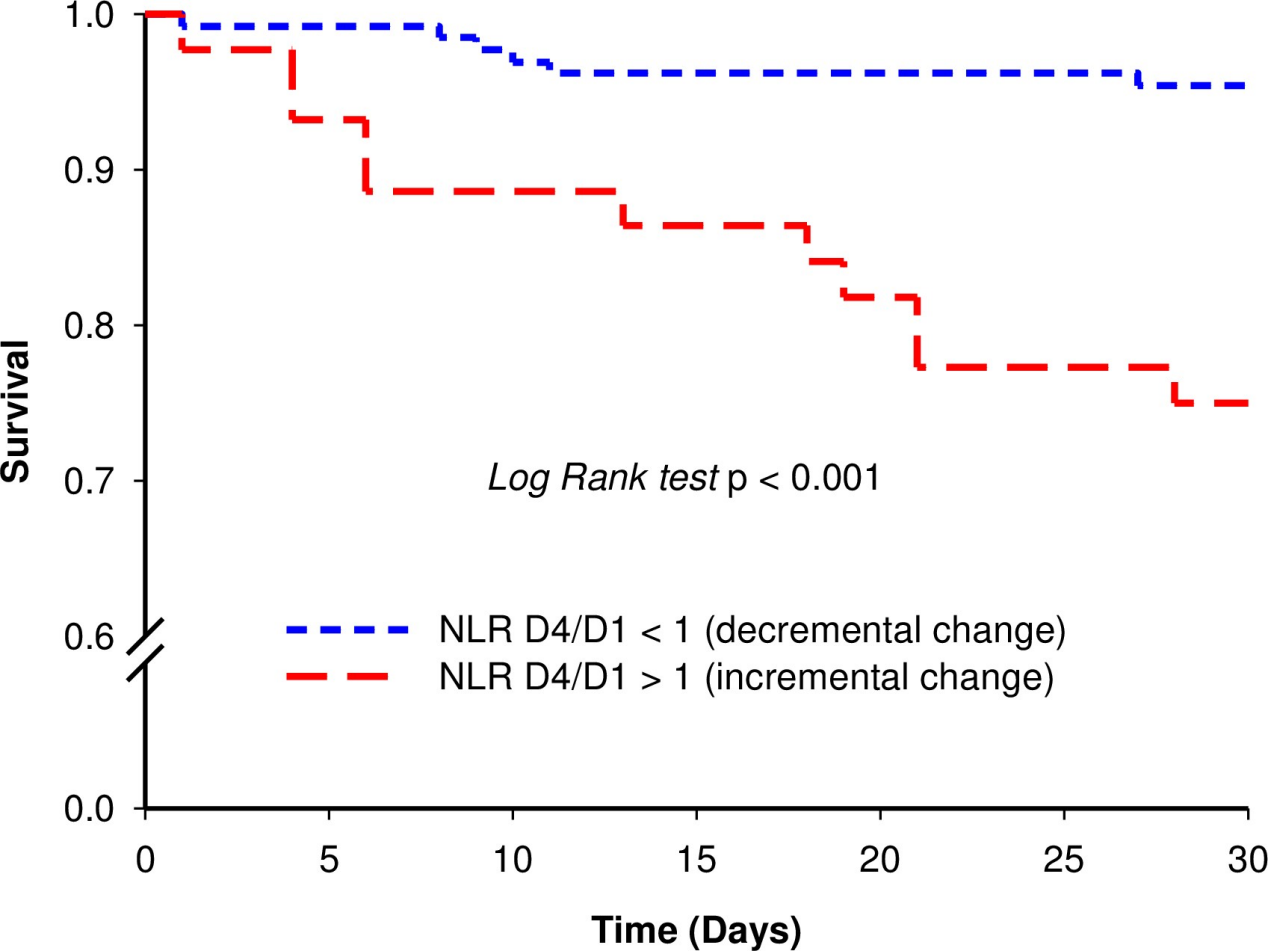
30-day survival curves according to incremental or decremental NLR change.

Multivariate regression analysis revealed that NLR D4, incremental NLR change (NLR D4/D1 > 1), PSI class, SOFA score, ICU admission, and clinical instability on day 4 were significant predictors of 30-day mortality (Table 4, Fig 2). In addition, there was a significant gradient of 30-day mortality between incremental and decremental NLR change (*Log Rank P* < 0.001) (Fig 3).

**Table 4:** Prognostic value of several variables for 30-day mortality.

	No adjustment		Adjustment^a^	
	Odds ratio (95% CI)	p value	Odds ratio (95% CI)	p value
Age	1.08 (1.02–1.14)	0.006	1.04 (0.98–1.10)	0.201
Men	7.40 (0.96–57.42)	0.055		
Cardiovascular disease	1.37 (0.42–4.50)	0.605		
Respiratory diseases*	2.77 (0.94–8.15)	0.065		
Diabetes mellitus	0.25 (0.03–1.93)	0.182		
Malignant disease	2.55 (0.75–8.72))	0.135		
Liver disease	1.20 (0.14–10.30)	0.869		
Chronic kidney disease	0.00 (0.00)	0.999		
NLR D1	1.01 (0.97–1.04)	0.640		
NLR D4	1.12 (1.06–1.18)	<0.001	1.11 (1.04–1.18)	0.003
NLR D4/D1	1.00 (0.96–1.05)	0.964		
NLR D4/D1 > 1	6.94 (2.39–20.17)	<0.001	7.10 (2.19–23.06)	0.001
CRP D1	1.03 (0.99–1.08)	0.156		
CRP D4	1.08 (1.02–1.14)	0.010	1.07 (1.00–1.14)	0.057
CRP D4/D1	0.91 (0.64–1.30)	0.602		
CRP D4/D1 > 1	2.14 (0.76–5.99)	0.149		
PCT D1	1.02 (0.99–1.04)	0.257		
PCT D4	1.12 (1.03–1.23)	0.011	1.09 (0.99–1.19)	0.076
PCT D4/D1	0.99 (0.83–1.18)	0.993		
PCT D4/D1 > 1	1.52 (0.43–5.31)	0.516		
PSI class	2.97 (1.63–5.40)	<0.001	2.41 (1.19–4.90)	0.015
SOFA score on admission	3.45 (1.98–6.02)	<0.001	4.23 (1.91–9.34)	<0.001
ICU admission	15.93 (5.20–48.85)	<0.001	11.29 (2.95–43.30)	<0.001
Clinical unstability on day 4	34.82 (7.53–160.95)	<0.001	21.61 (4.36–107.17)	<0.001

CI, confidence interval; CRP, C-reactive protein; ICU, intensive care unit; NLR, neutrophil-lymphocyte ratio; PCT, procalcitonin; PSI, pneumonia severity index; SOFA: sequential organ failure assessment.

^a^Adjusted the models for the age, sex, and PSI class.

### Prognostic biomarkers for early treatment response

Multivariate regression analysis demonstrated NLR D4 (odds ratio (OR): 1.09; 95% confidence interval (CI): 1.02–1.17; *P* = 0.009), incremental NLR change (OR: 18.02; 95% CI: 4.62–71.92; *P* < 0.001), and CRP D4 (OR: 1.08; 95% CI: 1.01–1.16; *P* = 0.027) were significant predictors of ICU admission. In addition, NLR D4 (OR: 1.12;95% CI: 1.06–1.19; *P* < 0.001), NLR D4/D1 (OR: 3.93; 95% CI: 1.96–7.88; *P* < 0.001), incremental NLR change (OR: 8.30; 95% CI: 3.25–21.20; *P* < 0.001), CRP D4 (OR: 1.07;95% CI: 1.01–1.13; *P* = 0.017), and incremental CRP changes (OR: 2.80;95% CI: 1.18–6.64; *P* = 0.020) were significant predictors of clinical unstability on day 4.

### Prognostic ability of NLR added to PSI

**Fig 4:**
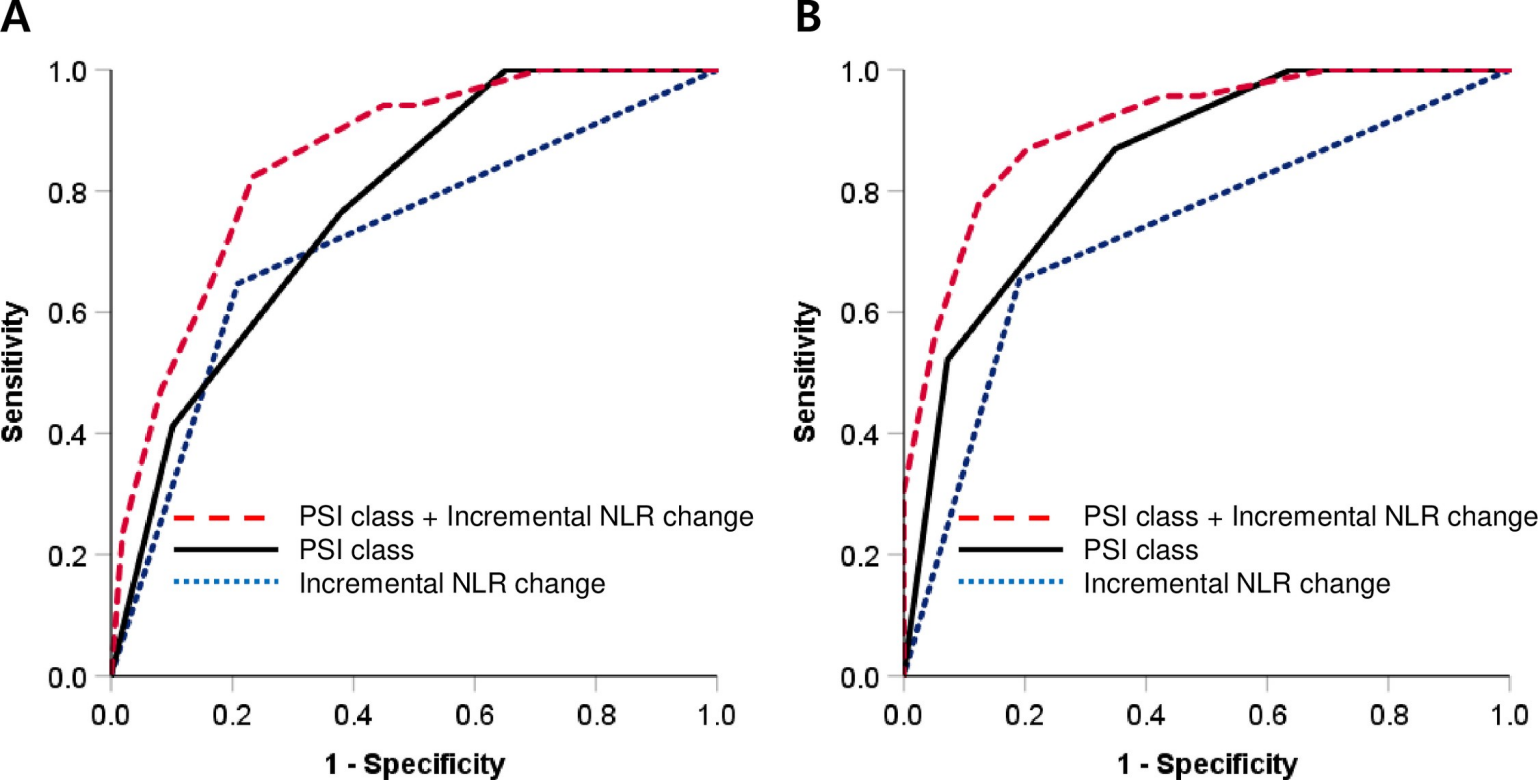
Receiver operating characteristic curve analyses of the ability of incremental NLR change (NLR D4/D1 > 1) and the pneumonia severity index class to predict 30-day mortality (A) and intensive care unit admission (B) in hospitalized patients with community-acquired pneumonia. The area under the curves significantly increased when incremental NLR change was added to the PSI class.

The ROC curve analyses of the ability of incremental NLR change and the PSI class to predict poor clinical outcomes are presented in Fig 4. The AUCs (95% CI) of incremental NLR change and the PSI to predict 30-day mortality were 0.719 (0.646–0.784) and 0.773 (0.704–0.833). The AUC increased significantly when incremental NLR change was added to PSI (0.853 (0.792–0.902) vs. 0.773 (0.704–0.833); *P* = 0.026). The AUC increased further when incremental NLR change was added to PSI compared with the addition of incremental CRP change to PSI (0.853 (0.792–0.902) vs. 0.796 (0.729–0.853); *P* = 0.118). The AUC predicting ICU admission also significantly increased when incremental NLR change was added to PSI (0.906 (0.853–0.945) vs. 0.844 (0.781–0.894); *P* = 0.039). In addition, the AUC increased further when incremental NLR change was added to PSI than when incremental CRP change was added to PSI (0.906 (0.853–0.945) vs. 0.863 (0.803–0.910); *P* = 0.137).

## Discussion

In this consecutive series of hospitalized CAP patients from real-world practice, serial NLR measurement was a powerful laboratory tool used to predict the prognosis and early treatment response. This study yielded following results. First, the NLR value on day 4 and incremental NLR change (NLR D4/D1 > 1) were significantly associated with 30-day mortality, ICU admission, and clinical unstability on day 4. Second, incremental NLR change was a significant predictor of 30-day mortality and ICU admission. Third, addition of incremental NLR change significantly improved the prognostic ability of the PSI. Fourth, the predictive ability of NLR for 30-day mortality or ICU admission was greater than that of CRP.

The circulating leukocyte count in infectious disease varies with time. Lymphocytopenia and neutrophilia are physiological reactions of the innate immune system to systemic inflammatory phenomenon particularly when caused by bacterial infection. Lymphocytopenia is caused by enhanced apoptosis and migration of lymphocytes within the liver, spleen and reticuloendothelial system and by lymphocyte redistribution within the lymphatic system [19, 20]. Neutrophilia is the opposite condition during systemic inflammatory response and is attributed to delayed apoptosis and stimulation of stem cells by several growth factors. The NLR integrates the two WBC subtypes with opposite reactions with regard to systemic inflammation. Accordingly, this ratio can be more powerful than either parameter alone. Several studies have shown that the NLR has greater prognostic power than traditional markers for infection such as WBC count, neutrophil count, and CRP in adult CAP patients [16, 21]. Another study has demonstrated that the NLR improves the accuracy and sensitivity of PSI to predict 30-day mortality in adult CAP patients [15]. In contrast, the NLR was not comparable to CRP and PCT for diagnosing or evaluating the severity of pneumonia in recently published study [22]. However, this study evaluated patients with hospital acquired pneumonia and analyzed the levels of NLR, CRP and PCT once measured on admission to the ICU. Recent Italian study have demonstrated that COVID-19 patients showed a significant reduction in admission WBC count and relatively more reduced neutrophil count than lymphocyte count compared with non-COVID-19 CAP patients [23]. In that study, authors claimed that WBC count could provide a simple and rapid tool for prompt COVID-19 diagnostic triage in CAP patients. This finding suggests that there might be room for the use of the NLR value in the early recognition of patient with COVID-19 pneumonia during COVID-19 outbreak.

To the best of our knowledge, this study is the first to demonstrate the clinical value of serial NLR measurement for assessing the prognosis and early treatment response in hospitalized CAP patients. In our study, the NLR value on day 4 and incremental NLR change between admission day and day 4 were the most powerful predictive inflammatory biomarkers. However, the levels of NLR, CRP, and PCT measured on admission did not differ significantly between survivors and non-survivors, suggesting that the initial levels do not facilitate mortality prediction in hospitalized CAP patients. This finding is in agreement with the results of recent studies evaluating CRP and PCT as biomarkers. Guo et al. showed that serial CRP and PCT levels rather than initial CRP and PCT levels had moderate predictive value for 30-day mortality and initial treatment failure in hospitalized CAP patients, so the dynamic CRP and PCT changes can be used to predict hospitalized CAP prognosis [11]. Another similar study by Ito et al. also demonstrated that only serial PCT measurement was useful to predict 30-day mortality and early treatment failure in hospitalized CAP patients [13]. Furthermore, the ability predicting 30-day mortality was improved by the combination of incremental PCT change (PCT D3/D1 > 1) and PSI. However, the same study group (Ito et al.) reported the usefulness of initial CRP by demonstrating the role of high initial CRP level as well as incremental PCT change as prognostic variables in CAP patients [24]. The different results may be attributed to the differences in basic clinical characteristics of the enrolled patients. We speculate that the short lifespan of neutrophil (around 7 h) and brief steady kinetics of neutrophil may have an effect on the variable prognostic value of NLR measured on admission. This phenomenon indicates the varying prognostic ability of NLR measured on admission depending on the duration and severity of CAP at the time of admission. Therefore, serial NLR measurement rather than one-time measurement is needed to assess the prognosis and early treatment response. The combination of incremental NLR change and PSI increases the clinical significance of the present study. Therefore, serial NLR measurement is essential not only to predict prognosis, but also to improve the predictive ability of the PSI. Our study also demonstrated that the prognostic value of NLR was greater than that of CRP. CRP is an acute phase reactant synthesized in the liver but is not a specific marker of infection. However, CRP has a lagging phase behind WBC and as a result NLR. The half-life of CRP is about 18–19 hours and is constant under all disease conditions, so plasma CRP level is dependent on how much was made and when, not how fast is being eliminated. Therefore, CRP value would decrease toward baseline level about 3 days after CAP-induced inflammatory response has recovered. This means that NLR would better reflect the change of CAP-induced inflammatory reactions over a short period of time (between admission day and hospital day 4) than CRP. In fact, NLR D4 and incremental NLR change (NLR D4/D1 >1) were significant predictors of 30-day mortality, but CRP D4 and incremental CRP change did not in our analyses. Several studies have reported controversial results regarding the role of CRP for the prediction of CAP prognosis [7–9, 11]. Serial change rather than initial measurement was of higher prognostic value even in studies showing the prognostic ability of CRP [9, 11]. NLR measurement costs less than CRP measurement, so it would be more useful to measure NLR serially than CRP for predicting prognosis and assessing early treatment response.

This study has several limitations. First, this was a non-randomized and retrospective observational single center study that did not include a large number of patients. Therefore, the possibility of selection bias or residual confounding from unknown or unmeasured covariates cannot be excluded. Second, this study did not have the validation group which might improve the reliability of our findings. We are planning a prospective observational study recruiting a larger population who will be randomized into the test cohort or the validation cohort. Third, when we are performing multivariate analysis, there is a high risk of random statistical significance. So, we did multivariate analysis adjusting the models for age, sex and PSI class which incorporated co-morbid illnesses, findings on vital signs and physical examination, and essential laboratory findings to reduce random effects as much as possible. Fourth, the data for serial measurements of PCT, a potent infection marker, was missed in a considerable number of patients, so we did not compare the prognostic value of serial measurement between NLR and PCT. Prospective studies comparing NLR, CRP, and PCT levels are warranted. Fifth, we presented incremental changes for serum inflammatory markers on day 1 and day 4. Other researchers may argue that another day would be better than day 4. However, the clinical response to therapy is usually evaluated on 3 to 4 days after treatment initiation in hospitalized CAP patients. Prospective studies are warranted to decide optimal measurement time to see interval change for serum inflammatory markers. Lastly, only hospitalized patients with a relatively large proportion of severe pneumonia (30-day mortality rate 9.7%) were included. Therefore, our results cannot be applicable to outpatients with CAP. In conclusion, it would be very helpful to measure the NLR serially not only on admission but also on hospital day 4 to predict the prognosis and to assess early treatment response in hospitalized CAP patients. Additionally, serial NLR measurement significantly improves the prognostic ability of the PSI.

## Supporting information

S1 File (SAV)
